# Disseminated Tuberculosis Presenting as Venous Thromboembolism

**DOI:** 10.7759/cureus.35575

**Published:** 2023-02-28

**Authors:** Ujjawal K Shriwastav, Mayank Agarwal, Bishal Shah, Shyam M Bohare, Monika Pathania

**Affiliations:** 1 Internal Medicine, All India Institute of Medical Sciences, Rishikesh, Rishikesh, IND

**Keywords:** pericarditis, pulmonary embolism, venous thromboembolism, virchow’s triad, tuberculosis

## Abstract

Tuberculosis (TB) is one of the leading causes of morbidity and mortality throughout the world and can have both pulmonary and extrapulmonary manifestations. Among the myriad extrapulmonary manifestations of TB, deep vein thrombosis (DVT) is rare. We present the case of a 25-year-old woman who presented with progressive painful swelling of the left upper limb associated with intermittent low-grade fever. Upon evaluation, she was found to have DVT along with a subsegmental pulmonary embolism. Further workup of the patient revealed bilateral pleural effusion and constrictive pericarditis along with microbiological evidence of *Mycobacterium tuberculosis*. The patient was started on anti-tubercular therapy along with therapeutic anti-coagulation, after which there was a substantial clinical improvement. Though rare, this case elucidates the venous thrombosis risk associated with one of the most common diseases in developing countries.

## Introduction

Tuberculosis (TB) is the 13th leading cause of death worldwide and the second major infectious killer after COVID-19, with an incidence of 10.6 million people in the year 2021 [1]. It has been the main culprit leading to significant morbidity and mortality in developing countries like India. Despite being rare, active or disseminated TB has been suggested to be an emerging risk factor for deep vein thrombosis (DVT), with a prevalence of around 1.5-3.4% as found in several studies [2].

## Case presentation

A 25-year-old woman without any known addictions and comorbidities presented to a tertiary care center with insidious onset, painful, progressive swelling of the left upper limb for two weeks associated with intermittent low-grade fever during the evening hours. The swelling was not associated with any history of trauma, immobilization or cannulation/instrumentation in the recent past. Detailed history revealed that fever was associated with periods of non-productive cough for six months, subsiding with over-the-counter medicines but without substantial improvement. There was no history of shortness of breath, chest pain, jaundice, decreased urine output, froth in the urine, joint pain, or recurrent abortions in the past. She lived in a joint family, had a childbirth one year back and gave no history of oral contraceptive intake. However, there is a history of contact with a case of active TB under anti-tubercular therapy in the family.

On examination, she was febrile, pale, thin-built and hemodynamically stable. The left upper limb was swollen, and tender with a limited range of active motion. There was no redness or pus discharge. Peripheral pulses were present. A general physical examination did not reveal any obvious or significant lymphadenopathy, and a breast exam was normal. On auscultation of the chest, there were decreased breath sounds in the bilateral basal regions.

**Figure 1:**
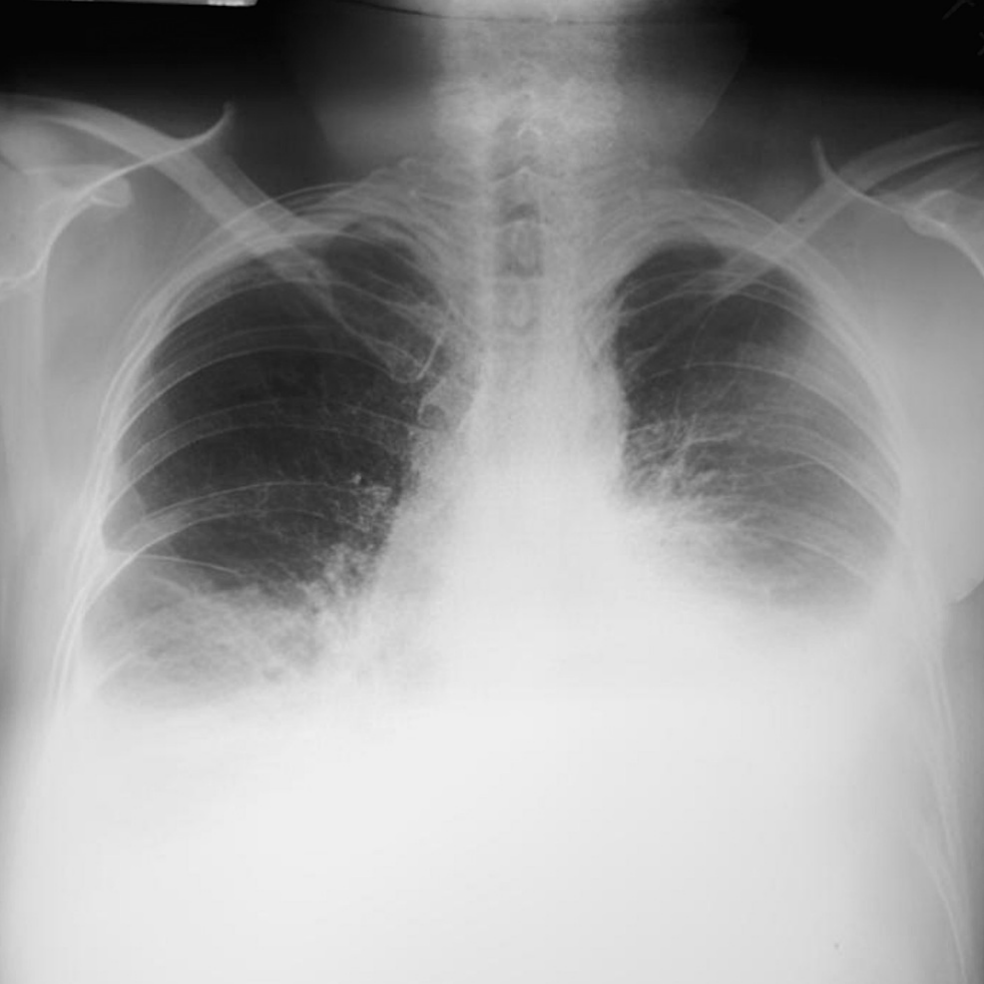
Chest x-ray PA view shows blunting of the bilateral costophrenic angle, suggestive of bilateral pleural effusion. PA: postero-anterior.

**Figure 2:**
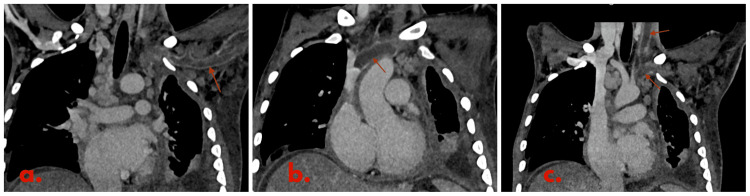
Coronal venous phase CT-image showing a hypodense non-enhancing filling defect causing non-opacification of the left visualized distal axillary vein (a), left subclavian vein (b), left internal jugular vein and left brachiocephalic vein and extension into the SVC (c). SVC: superior vena cava.

**Figure 3:**
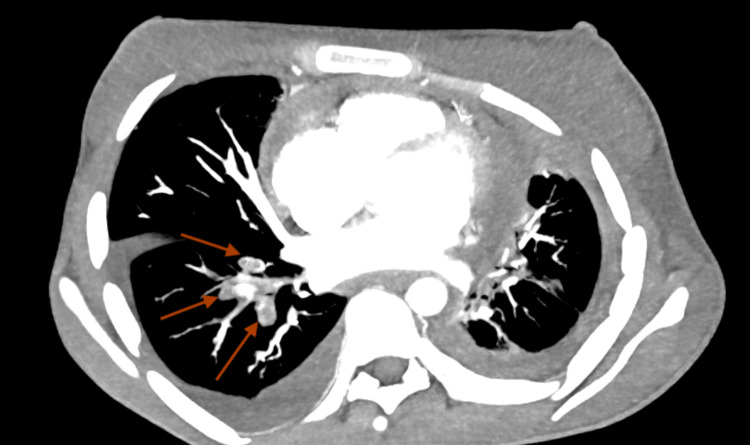
Axial arterial phase image-non-enhancing filling defect of the segmental branches of the right lower pulmonary artery.

**Figure 4:**
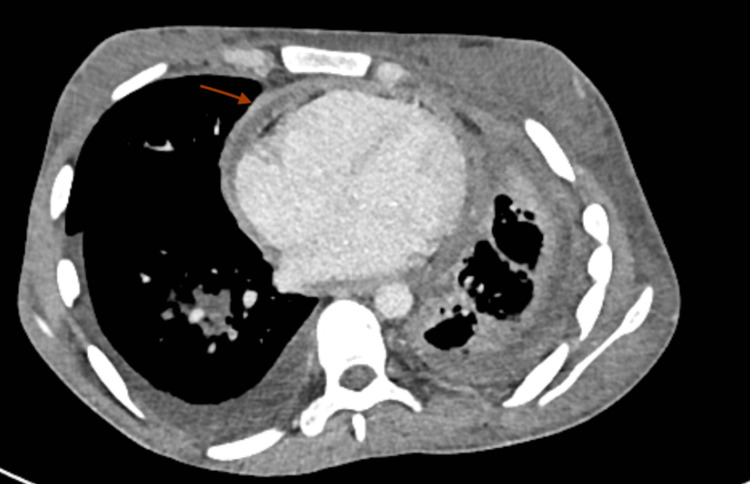
Axial contrast image of the thorax showing enhancing pericardial thickening with pericardial effusion suggestive of pericarditis.

On further evaluation, the hemogram showed moderate anemia (Table 1). The chest x-ray was suggestive of bilateral pleural effusion (Figure 1). A color doppler scan of the left upper limb showed an echogenic thrombus within the left internal jugular vein, subclavian and axillary vein completely occluding the lumen without any color flow or compressibility. The right upper limb and bilateral lower limb color doppler scans were negative for any thrombus. Thrombophilia workup was negative with normal protein C, protein S, and anti-thrombin III levels. Antinuclear antibodies (ANA) and antiphospholipid antibodies (APLA) profiles were also negative for the patient (Table 2). 2D echocardiography showed right ventricular dysfunction along with features of constrictive pericarditis. A CT-pulmonary angiography was obtained that showed thromboembolism of subsegmental branches of the right lower pulmonary artery along with minimal left pleural effusion (Figures 2, 3). Noticeable and significant mediastinal lymphadenopathy was also seen. The CT chest also shows the presence of pericardial effusion with thickened pericardium suggestive of pericarditis (Figure 4).

**Table 1:** Investigations. ADA: adenosine deaminase, ALP: alkaline phosphatase, DLC: differential leukocyte count, GGT: gamma-glutamyl transferase, INR: international normalized ratio, LDH: lactate dehydrogenase, aPTT: activated partial thromboplastin clotting time, SGOT: serum glutamic-oxaloacetic transaminase, SGPT: serum glutamic pyruvic transaminase, TLC: total leucocyte count.

Investigations							
	16/6/2022	17/06/2022	19/06/2022	23/06/2022	25/06/2022	28/06/2022	Reference value
Hb (g/dl)	8.7		9.323		9.6		13-17
TLC (×1000/μl)	12.24		11.27		10.33		4-11
Platelet (×1000/μl)	292.4		415.8		554		150-400
DLC (N/L/M/E)	78.3/11.19/7.21/1.9		78.8/11.41/6.28/1.75		76.2/12.3/8.8/2.1		N (40-70)%, L (20-40)%, M (2-8)%, E (1-6)%
Total bil/direct bil (mg/dl)		1.15/0.36			0.87/0.19	0.74/0.36	Total bil: 0.3-1.2, direct bil: 0-0.2
SGPT (U/L)		22			20	15	0-35
SGOT (U/L)		46			27	20	0-35
ALP (U/L)		162			165	177	30-120
GGT (U/L)		45			50	51	0-38
Total protein (g/dl)		5.6			6.4	5.3	6.6-8.3
Albumin (g/dl)		2.4			3.8	2.6	3.5-5.2
Urea (mg/dl)	18		21	27			17-43
Creatinine (mg/dl)	0.37		0.64	0,74			0.72-1.18
Na (mmol/L)	130		137	135			136-146
Potassium (mmol/L)	3.93		3.5	3.5			3.5-5.1
Calcium (mg/dl)	8.11		8.7	7.7			8.8-10.6
Uric acid (mg/dl)	5.0		6.7	5.6			3.5-7.2
Phosphorous (mg/dl)	2.9		4	5			2.5-4.5
PT/INR	15.9/1.24						
aPTT	24.2						
HbA1c	5.3%						5.7-6.4%
Serum LDH (U/L)			266				
Homocysteine (μmol/l)			9.45				5-15
D-dimer (mcg/ml)	3.25						<0.5

**Table 2:** Other investigations. LDH: lactate dehydrogenase, ADA: adenosine deaminase, CB-NAAT: cartridge-based nucleic acid amplification test, ANA: antinuclear antibodies, DRVVT: diluted Russell viper venom time.

Other investigations			
			Reference value
Pleural fluid analysis	Total cells	140 cells/μl	
	Sugar	119.9 mg/dl	
	Protein	2.9 g/dl	
	LDH	208 U/L	
	ADA	9.9	
	CB-NAAT	Positive with no resistance to rifampicin	
	Total cells	140 cells/μl	
	Sugar	119.9 mg/dl	
Thrombophilia profile			
	Protein C, functional	100.00%	70-140
	Protein S, free	94.00%	60-140
	Anti-thrombin activity, functional	100.00%	80-120
ANA on Hep 2 cells		<1:80	
Antiphospholipid antibody panel			
	Beta 2 glycoprotein, IgG (serum)	2.20 SGU	<20.00
	Beta 2 glycoprotein, IgM (serum)	6.18 SMU	<20.00
	Cardiolipin antibody, IgG (serum)	8.87 GPL	<15.00
	Cardiolipin antibody, IgM (serum)	9.92 MPL	<12.50
Lupus anticoagulant by DRVVT	Patient value	35.40 s	31.60-41.00
	Control value	36.30	
	Screen ratio	0.97	
	Normalized ratio	<1.20	

Ultrasound-guided diagnostic pleural effusion tapping was done, which was exudative along with positive for acid-fast bacilli in modified Ziehl-Neelsen staining. Rifampicin-sensitive *Mycobacterium tuberculosis* was detected on the cartridge-based nucleic acid amplification test (CB-NAAT). The patient was initiated on anti-coagulation and weight-based anti-tubercular therapy (ATT). Subsequently, there was a gradual resolution in swelling and fever spikes. The patient was later discharged on anti-tubercular therapy along with dabigatran. She has been counseled to avoid pregnancy for the period of anti-coagulation. She has been planning to continue anti-TB treatment for six months from the directly observed treatment, short course (DOTS) center as per national guidelines, along with anti-coagulation for the same duration. On follow-up after four weeks, there was complete resolution in swelling and the fever had subsided.

## Discussion

Disseminated TB is defined as the involvement of two or more non-contagious sites/organ systems due to the lympho-hematogenous spread of *Mycobacterium tuberculosis*. Epidemiological data have suggested that one-fourth or more cases of TB are extra-pulmonary, out of which less than almost 20% of cases are disseminated TB [3]. The exact mechanism of dissemination of *Mycobacterium tuberculosis* is not clear, but it has been suggested that the erosion of epithelial cells of alveoli leads to entry to bacteria into pulmonary veins resulting in dissemination [4].

The study of various reports on common clinical manifestations and organ system involvement in a case of disseminated TB suggests that constitution clinical features, lymphadenopathy, pleural effusion, and ascites top the list among various other manifestations [5]. The review of literature from the past has expounded active TB to be one of the emerging risk factors for the development of deep vein thrombosis, with a prevalence of around 1.5-3.4% [2].

The prevalence of venous thromboembolism (VTE) in the form of DVT and pulmonary embolism are underestimated as they are often silent despite being common. The pathogenesis of venous thrombosis is explained by Virchow’s triad, consisting triad of hypercoagulability, venous stasis, and endothelial injury [6]. Among the numerous risk factors that have been explained for the development of venous thrombosis, infection is one of them. Sepsis is an example of systemic inflammation with organ dysfunction, where disseminated intravascular coagulation has been observed as a major complication and cause of death. Coagulation activation and immunothrombosis have been considered hallmarks of sepsis [7].

Apart from sepsis and hospital settings, similar associations between infection and thrombus formation can be found in a community setting [8]. One example of a community-acquired infection is TB, which is caused by the acid-fast bacilli *Mycobacterium tuberculosis*. There have been several mechanisms to explain venous thromboembolism in TB, like the induction of inflammation, the transient hypercoagulable state, and direct endothelial injury. TB is a pro-inflammatory state that has been associated with an increase in acute phase reactants like elevated plasma fibrinogen, which when coupled with a decrease in anti-thrombin III appears to support the development of DVT in TB [9]. Thus, TB has been described as one of the hypercoagulable states with enhanced activation of coagulation along with altered mechanisms of anticoagulation [10]. There have been reports to suggest that TB can result in the development of anti-phospholipid antibodies, ensuing thrombosis of vessels [11]. Another widely accepted mechanism for the development of venous thrombosis in active TB has been put forward on account of the use of rifampicin in the first-line treatment of TB [12]. Our case has been a classical example of community-acquired infection-related thrombosis of large veins. After ruling out all the other causes of thrombosis in a young female, we concluded that disseminated TB led to DVT in this case. In cases with the presence of transient risk factors, like TB in this case, the management of DVT consists of anti-coagulation for a short course of three to six months [13].

## Conclusions

Among myriad clinical manifestations of disseminated TB, deep venous thrombosis and pulmonary embolism could be the presenting manifestations. Despite the prevalence is very low, there have been many case reports explaining the induced coagulation and suppressed anti-coagulation resulting in VTE in cases of active TB. Early identification and anticipation of such complications with timely initiation of treatment, especially in resource-poor settings, can result in a significant decrease in the mortality and morbidity associated with them.

## References

[1] (2022). Global tuberculosis report. https://www.who.int/publications/i/item/9789240037021.

[2] Gupta A, Mrigpuri P, Faye A, Bandyopadhyay D, Singla R (2017). Pulmonary tuberculosis-an emerging risk factor for venous thromboembolism: a case series and review of literature. Lung India.

[3] Sharma SK, Mohan A, Sharma A (2016). Miliary tuberculosis: a new look at an old foe. J Clin Tuberc Mycobact Dis.

[4] Krishnan N, Robertson BD, Thwaites G (2010). The mechanisms and consequences of the extra-pulmonary dissemination of Mycobacterium tuberculosis. Tuberculosis.

[5] Bagot CN, Arya R (2008). Virchow and his triad: a question of attribution. Br J Haematol.

[6] Khan FY (2019). Review of literature on disseminated tuberculosis with emphasis on the focused diagnostic workup. J Fam Community Med.

[7] Tani VM, Assis-Mendonça GR, da Silva TB, Rogerio F, De Paula EV (2017). Microvascular thrombosis in sepsis: an autopsy study. Thromb Res.

[8] Smeeth L, Cook C, Thomas S, Hall AJ, Hubbard R, Vallance P (20061). Risk of deep vein thrombosis and pulmonary embolism after acute infection in a community setting. Lancet.

[9] Suárez Ortega S, Artiles Vizcaíno J, Balda Aguirre I, Melado Sánchez P, Arkuch Saade ME, Ayala Galán E, Betancor León P (1993). Tuberculosis as risk factor for venous thrombosis. Ann Med Interna.

[10] Kager LM, Blok DC, Lede IO (2015). Pulmonary tuberculosis induces a systemic hypercoagulable state. J Infect.

[11] Robson SC, White NW, Aronson I, Woollgar R, Goodman H, Jacobs P (1996). Acute-phase response and the hypercoagulable state in pulmonary tuberculosis. Br J Haematol.

[12] White NW (1989). Venous thrombosis and rifampicin. Lancet.

[13] Ortel TL, Neumann I, Ageno W (2020). American Society of Hematology 2020 guidelines for management of venous thromboembolism: treatment of deep vein thrombosis and pulmonary embolism. Blood Adv.

